# Midlife Cardiovascular Fitness Is Reflected in the Brain's White Matter

**DOI:** 10.3389/fnagi.2021.652575

**Published:** 2021-04-06

**Authors:** Tracy d'Arbeloff, Maxwell L. Elliott, Annchen R. Knodt, Maria Sison, Tracy R. Melzer, David Ireland, Sandhya Ramrakha, Richie Poulton, Avshalom Caspi, Terrie E. Moffitt, Ahmad R. Hariri

**Affiliations:** ^1^Department of Psychology & Neuroscience, Duke University, Durham, NC, United States; ^2^New Zealand Brain Research Institute, Christchurch, New Zealand; ^3^Department of Medicine, University of Otago, Christchurch, New Zealand; ^4^Dunedin Multidisciplinary Health and Development Research Unit, Department of Psychology, University of Otago, Dunedin, New Zealand; ^5^Social, Genetic, & Developmental Psychiatry Research Centre, Institute of Psychiatry, Psychology, & Neuroscience, King's College London, London, United Kingdom; ^6^Department of Psychiatry & Behavioral Sciences, Duke University School of Medicine, Durham, NC, United States; ^7^Center for Genomic and Computational Biology, Duke University, Durham, NC, United States

**Keywords:** Alzheimer's disease, aging, white matter, cardiovascular fitness, fitness behavior, healthy lifestyle, neurodegeneration

## Abstract

Disappointing results from clinical trials designed to delay structural brain decline and the accompanying increase in risk for dementia in older adults have precipitated a shift in testing promising interventions from late in life toward midlife before irreversible damage has accumulated. This shift, however, requires targeting midlife biomarkers that are associated with clinical changes manifesting only in late life. Here we explored possible links between one putative biomarker, distributed integrity of brain white matter, and two intervention targets, cardiovascular fitness and healthy lifestyle behaviors, in midlife. At age 45, fractional anisotropy (FA) derived from diffusion weighted MRI was used to estimate the microstructural integrity of distributed white matter tracts in a population-representative birth cohort. Age-45 cardiovascular fitness (VO_2_Max; *N* = 801) was estimated from heart rates obtained during submaximal exercise tests; age-45 healthy lifestyle behaviors were estimated using the Nyberg Health Index (*N* = 854). Ten-fold cross-validated elastic net predictive modeling revealed that estimated VO_2_Max was modestly associated with distributed FA. In contrast, there was no significant association between Nyberg Health Index scores and FA. Our findings suggest that cardiovascular fitness levels, but not healthy lifestyle behaviors, are associated with the distributed integrity of white matter in the brain in midlife. These patterns could help inform future clinical intervention research targeting ADRDs.

## Introduction

An aging global population has highlighted the need to preserve and prolong both physical and mental health to slow the accumulating social and financial burden associated with extended longevity (Burns et al., 2008; Christensen et al., 2009; Deary et al., 2009; Dougherty et al., 2017; Chang et al., 2019). This burden, in part, reflects increased numbers of older adults with Alzheimer's Disease and Related Dementias (ADRD) including vascular dementia and frontotemporal dementia. Recent estimates suggest that more than 5 million Americans aged 65 years and older are currently affected by ADRD (Facts and Figures, 2020). However, no cure for ADRD currently exists. Thus, there is a critical need for research into preventative measures or interventions to delay or prevent onset of ADRD and, more generally, minimize the impact of aging on physical and mental health.

Of particular importance to this goal are large-scale efforts to identify effective early interventions against age-related deterioration of brain structure, which precedes clinical diagnosis of ADRD and is often a precursor to decreased quality of life and cognitive decline (Freedman et al., 2002; Deary et al., 2009; Bennett and Madden, 2014; Brasure et al., 2018; Ding et al., 2018; Fan et al., 2019; Musiek and Morris, 2020; Tarumi et al., 2020). One potential intervention against aging-related structural decline in the brain is improving aspects of physical health, such as cardiovascular fitness (Cyarto et al., 2012; Zhu et al., 2015; Boraxbekk et al., 2016; Voss et al., 2016; Matura et al., 2017; Ding et al., 2018; Halloway et al., 2018; Clark et al., 2019; Wassenaar et al., 2019; Johnson et al., 2020; Tarumi et al., 2020). As a measure of the maximum rate at which the body can utilize oxygen (Garatachea et al., 2015; Beltz et al., 2016; Voss et al., 2016; Harridge and Lazarus, 2017), cardiovascular fitness reflects how efficiently the respiratory and circulatory systems are providing oxygenated blood to the body during active moments (Beltz et al., 2016; Williams et al., 2017). The advantages of good cardiovascular fitness for physical health are well-documented, including increased mobility, increased quality of life, and decreased cardiovascular disease risk (Etnier et al., 2006; Evans, 2010; Erickson et al., 2014; Harridge and Lazarus, 2017). Importantly, emerging evidence suggests cardiovascular fitness may also benefit structural brain integrity.

Better cardiovascular fitness has been associated with structural features of the brain's gray matter including greater average cortical thickness (Hurtz et al., 2014; Vuksanovi et al., 2019; Nicastro et al., 2020), total cortical surface area (Vuksanovi et al., 2019; Elliott, 2020), and subcortical volume (Erickson et al., 2011; Dougherty et al., 2017; Jonasson et al., 2017; Feter et al., 2018). We recently reported that midlife cardiovascular fitness was associated with thicker frontotemporal cortex and greater gray matter volume of cerebellar cortex in members of the Dunedin Study, which has followed a large population representative birth cohort for five decades. Importantly, aging-related cortical thinning in frontotemporal regions has been associated with cognitive decline in both healthy individuals and those with Alzheimer's disease (Singh et al., 2006; Burggren et al., 2008; McGinnis et al., 2011). Moreover, frontotemporal atrophy is a common archetype of pathological aging and is considered one of the main causes of dementia (Fjell et al., 2015; Cox et al., 2021). Thus, associations between cardiovascular fitness and frontotemporal cortical thickness suggest possible salubrious effects of improving fitness on age-related brain atrophy. That these associations were detectable in midlife is important as this is a window in the lifespan ripe for early targeted interventions to slow or even prevent age-related structural decline in the brain associated with risk for ADRD before too much damage has accrued (Sperling et al., 2014; Moffitt et al., 2017; Wassenaar et al., 2019).

Like gray matter, structural atrophy of white matter is also indicative of increased risk for ADRD and closely linked with cognitive ability (Au et al., 2006; Deary et al., 2006, 2009; Penke et al., 2012; Bennett and Madden, 2014; Cole and Franke, 2017; Mito et al., 2018; Fan et al., 2019; Elliott, 2020). In fact, research suggests that aging-related deterioration of the structural integrity of white matter may better signal later cognitive decline and mild cognitive impairment than gray matter, as white matter may be more susceptible to early aspects of disordered aging (Liu et al., 2017; Araque Caballero et al., 2018; Mito et al., 2018; Wen et al., 2019). However, comparably less research has been conducted on possible links between cardiovascular fitness and white matter integrity and what research does exist is a mix of positive, negative, and null findings (Perea et al., 2016; Sexton et al., 2016, 2020; Voss et al., 2016; Fissler et al., 2017; Clark et al., 2019; Wassenaar et al., 2019). As age-related atrophy of white matter tends to be more widespread than localized (Liu et al., 2017), one reason for the trend of mixed findings could be the focus on the microstructural integrity of individual white matter tracts rather than assessing overall integrity across the brain. Studies investigating connections between cardiovascular fitness and white matter, especially in younger or cognitively healthy cohorts where changes in white matter microstructural integrity may be less apparent (Liu et al., 2017), could overlook small distributed changes that may not survive correction for multiple comparisons within any one tract.

Another possible reason for the observed mixed results could be the conflation of cardiovascular fitness with healthy lifestyle behaviors such as physical activity. Lifestyle interventions designed to improve cardiovascular fitness often do so indirectly, such as by increasing physical activity (Sexton et al., 2016; Wassenaar et al., 2019; d'Arbeloff, 2020). However, increasing healthy lifestyle behaviors is not necessarily correlated with improved cardiovascular fitness (d'Arbeloff, 2020). Thus, prior studies using self-report measures of healthy lifestyle behaviors as a proxy for cardiovascular fitness when examining associations with white matter structural integrity may have yielded different results from studies using direct measures of cardiovascular fitness (Sexton et al., 2016; d'Arbeloff, 2020).

Here, we used data from members of the Dunedin Study to examine possible differential associations between distributed white matter integrity and both healthy lifestyle behaviors (*N* = 854) and cardiovascular fitness (*N* = 801) in midlife. As previous studies focusing on specific white matter tracts have yielded mixed findings (Perea et al., 2016; Sexton et al., 2016; Fissler et al., 2017; Clark et al., 2019; Wassenaar et al., 2019), we did not limit our analyses to *a priori* tracts of interest. Instead, we leveraged exploratory elastic net modeling to assess how cardiovascular fitness and healthy lifestyle behaviors were independently associated with distributed white matter integrity across the brain (Lee et al., 2015; Liu et al., 2017). Identifying differential links between cardiovascular fitness and healthy lifestyle behaviors with white matter integrity could help guide the optimal matching of putative interventions with midlife brain biomarkers in future clinical intervention research.

## Materials and Methods

### Study Design and Population

Data were derived from the Dunedin Study, a longitudinal investigation of health and behavior in a population representative birth cohort. Study members (*N* = 1,037; 91% of eligible births; 52% male) are all individuals born between April 1972 and March 1973 in Dunedin, New Zealand (NZ), who were eligible based on residence in the province and who participated in the first assessment at age 3 years (Poulton et al., 2015). The cohort represented the full range of socioeconomic status (SES) in the general population of NZ's South Island and as adults matched the NZ National Health and Nutrition Survey on key adult health indicators (e.g., body mass index (BMI), smoking, GP visits) and the NZ Census of citizens of the same age on educational attainment (Richmond-Rakerd et al., 2020). The cohort is primarily white (93%), matching South Island demographics (Poulton et al., 2015). Data were available at birth and assessments were carried out at ages 3, 5, 7, 9, 11, 13, 15, 18, 21, 26, 32, 38, and most recently (completed April 2019) 45 years, when 94.1% (*N* = 938) of the 997 participants still alive took part. Of these 938 Study members, 875 (93%) completed MRI scanning. Attrition analyses revealed that scanned Study members resembled still-living cohort members on childhood IQ and their family-of-origin's socio-economic status (Supplementary Figure 1). The relevant ethics committees approved each phase of the Study and written informed consent was obtained from all Study members before their participation.

### Estimated Maximum Oxygen Uptake

Midlife cardiovascular fitness was estimated by measuring heart rate in response to a submaximal exercise test on a friction-braked cycle ergometer at age 45. Depending on the extent to which heart rate increased during a 2-min 50W warm-up, the workload was adjusted to elicit a steady heart rate in the range of 130–170 beats per minute. After a further 6-min constant power output stage, the maximum heart rate was recorded and used to estimate maximal volume of oxygen uptake (VO_2_Max) adjusted for body weight in milliliters per minute per kilogram (mL/min/kg) according to standard protocols (Cullinane et al., 1988).

### Healthy Lifestyle Behaviors

Four major lifestyle factors (smoking history, average alcohol consumption, body mass index (BMI), and leisure-time physical activity) were combined into an aggregate measure of health and fitness behavior based on a recently published healthy lifestyle index (Nyberg et al., 2020). First, each of the four lifestyle factors were independently scored based on the following prespecified thresholds:

- BMI: <25.0 (2 points; optimal), 25.0–29.9 (1 point; intermediate), and ≥30.0 (0 points; poor)
- Smoking: Never smoked (2 points; optimal), former smoker (1 point; intermediate), and current smoker (0 points; poor)
- Average midlife alcohol consumption (total number of alcoholic drinks consumed in an average week; 1 drink equals 10 g of ethanol): 1–14 (women) or 1–21 (men) drinks per week (2 points; optimal), no alcohol consumed (1 point, intermediate), and ≥15 (women) or ≥22 (men) drinks per week (0 points; poor)
- Average midlife leisure-time physical activity: ≥2.5 h of moderate activity/week or ≥1.25 h of vigorous activity/week (2 points; optimal), activity levels lower than optimal but higher than poor (1 point; intermediate), and no or very little moderate/vigorous activity/week (0 points; poor).

The score for each factor was summed to compute an overall healthy lifestyle score (i.e., Nyberg Health Index) for each participant resulting in scores ranging from 0 (lowest healthy behaviors, highest risk for negative health outcomes) to 8 (highest healthy behaviors, lowest risk for negative health outcomes).

### MRI Data Acquisition and Processing

Each participant was scanned using a MAGNETOM Skyra (Siemens Healthcare GmbH) 3T scanner equipped with a 64-channel head/neck coil at the Pacific Radiology Group imaging center in Dunedin, NZ. Diffusion-weighted images providing full brain coverage were acquired with 2.5 mm isotropic resolution and 64 diffusion weighted directions (4,700 ms repetition time, 110.0 ms echo time, *b*-value 3,000 s/mm^2^, 240 mm field of view, 96 × 96 acquisition matrix, slice thickness = 2.5 mm). Non-weighted (b = 0) images were acquired in both the encoding (AP) and reverse encoding (PA) directions to allow for EPI distortion correction.

Diffusion images were processed in FSL (http://fsl.fmrib.ox.ac.uk/fsl) as follows. Raw diffusion-weighted images were corrected for susceptibility artifacts, subject movement, and eddy currents using topup and eddy. Images were then skull-stripped and fitted with diffusion tensor models at each voxel using FMRIB's Diffusion Toolbox (FDT; http://fsl.fmrib.ox.ac.uk/fsl/fslwiki/FDT). The resulting FA images from all participants were non-linearly registered to the FA template developed by the Enhancing Neuro Imaging Genetics Through Meta-Analysis consortium (ENIGMA), a minimal deformation target calculated across a large number of individuals (Jahanshad et al., 2013).

**Figure 1:**
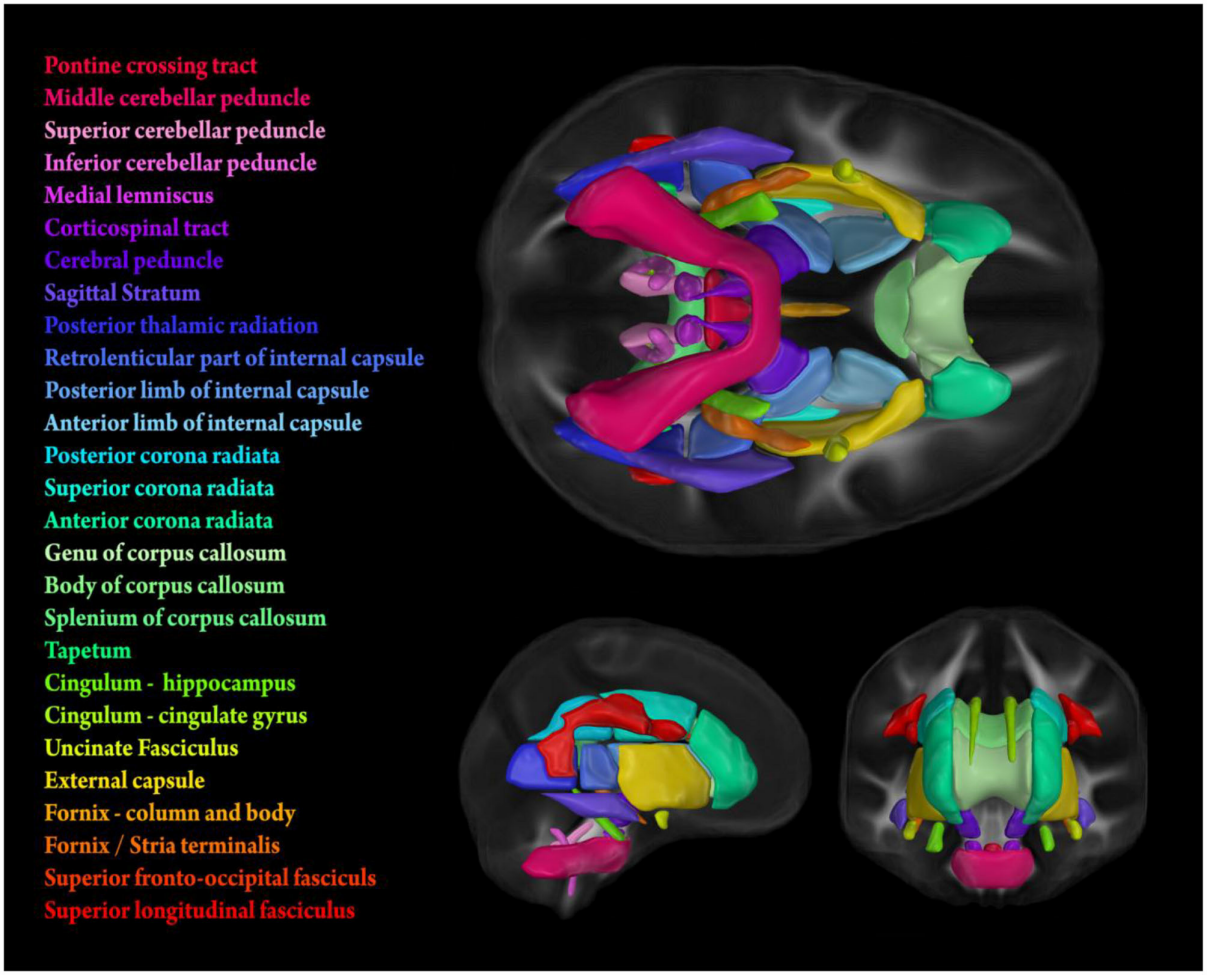
Stylized depiction of 21 bilateral and 6 midline tracts from the Johns Hopkins University (JHU) white matter parcellation atlas (Mori et al., 2005). FA values used for analyses were obtained by calculating tract-wise means from the intersection of the atlas and the individual FA skeletons generated using TBSS (see subsection MRI Data Acquisition and Processing for details).

The images were then processed using the tract-based spatial statistics (TBSS) (Smith et al., 2006) modified to project individual FA values onto the ENIGMA-DTI skeleton. Following the extraction of the skeletonized white matter and projection of individual FA values, ENIGMA-tract-wise regions of interest, derived from the Johns Hopkins University (JHU) white matter parcellation atlas (Mori et al., 2005), were overlaid to extract the mean FA across the full skeleton and average FA values for 21 bilateral and 6 midline tracts (i.e., 48 individual values, Figure 1). Data from 7 Study members were removed because diffusion images were collected with 20-channel head coil to accommodate large head and shoulder size, leading to poor diffusion image quality per visual inspection. In addition, data from 3 Study members were removed due to major incidental findings, 5 due to excessive (>3 mm) motion detected with the eddy tool, and 6 due to missing diffusion scans. Thus, there were high-quality diffusion imaging data from 854 Study members for the current analyses. Nyberg Health Index scores were calculated for all of these 854 Study members. An additional 53 subjects were missing estimated VO_2_Max due to a myriad of issues (e.g., disability, injury, non-compliance, failing to complete the task, and machine malfunction) leaving an *N* of 801 for cardiovascular fitness analyses.

### Analyses

The R package “caret” was utilized to run all analyses (Kuhn, 2008). To address sex differences in VO_2_Max (Supplementary Figure 2) and parse out the unique variance accounted for solely by distributed white matter integrity, we regressed out sex and ran all analyses using residualized scores. As there was no such stratification in Nyberg Health Index scores, sex was simply included as a covariate in analyses of this measure.

To avoid over-fitting and adjust for high correlations between variables, we used elastic net modeling, a regularized regression method that incorporates the mixed penalty term l_1_-norm (λ_1_) from Least Absolute Shrinkage and Selection Operator (LASSO) and the penalty term l_2_-norm (λ_2_) from Ridge regression. Elastic net, like LASSO and Ridge penalty regression methods, uses penalty terms to minimize both bias and variance in base ordinary least square (OLS) models through shrinking regression coefficients toward zero (Guo et al., 2018). However, studies have indicated that the use of elastic net can result in lower mean squared error than LASSO or Ridge when variables are highly correlated (Waldmann et al., 2013), as was the case with average FA values for the 48 white matter tracts used in our analyses (Avg *r* = 0.29, Range = 0.002–0.82; Supplementary Figure 3). Further, elastic net results in a higher number of correctly identified predictor variables than LASSO and has a lower false positive rate than Ridge (Zou and Hastie, 2005; Waldmann et al., 2013). An additional parameter, α, is used in elastic net to determine how much weight should be given to either λ_1_ or λ_2_. An elastic net with an α value of 0 performs much like Ridge regression; an α value of 1 performs like LASSO.

In our analyses, data were first randomly split into a training (70%) and a test (30%) subset. After centering and scaling all variables in the training subset, Ten-fold cross-validation was used to determine the best α and λ values that correspond to the lowest prediction error and the best model fit. Optimized parameters were then inserted into the model and the model was trained to predict sex-adjusted VO_2_Max within the training subset. Through the initial training, we obtained weighted partial coefficients for each of the predictors included in the model. We then used the trained model to predict scores in the held-aside test subset and generate predicted residualized values for each white matter tract. Prediction within the test subset was assessed via correlating actual and predicted residualized scores and calculating *R*^2^ and RMSE statistics.

To further ensure confidence in the robustness of our results and to minimize generalization error of the prediction, we used a form of ensemble modeling called bagging (Kotu and Deshpande, 2015). The full elastic net analyses described above were re-run for both variables of interest (residualized VO_2_Max and Nyberg Health Index) 1,000 times, each time with a new randomly split testing and training dataset. The ensemble model then aggregates the prediction of each of the 1,000 base models (Kotu and Deshpande, 2015). Distributions and means of output statistics of each of the base models were used to improve accuracy and confidence in the predictive capacity of our models and partial regression coefficients.

## Results

### Cohort Characteristics

The average VO_2_Max was 26.99 mL/min/kg (SD = 7.37, range = 9.05–48.76) and the average score on the Nyberg Health Index was 4.97 (SD = 1.84, range = 0–8). There was a significant association between VO_2_Max and Nyberg Health Index scores (β = 0.27, CI = 0.22–0.32, *p* < 0.001). For average FA values of each white matter tract used in the analyses see Supplementary Table 1. Test-retest reliability of tract-wise FA, determined using data from a subset of 20 Study members who were scanned a second time (Elliott et al., 2020), was high (mean ICC = 0.879 ± 0.109 SD). Information on inter-tract correlations can be seen in Supplementary Figure 3.

### Elastic Net Modeling of Cardiovascular Fitness (*N* = 801)

**Figure 2:**
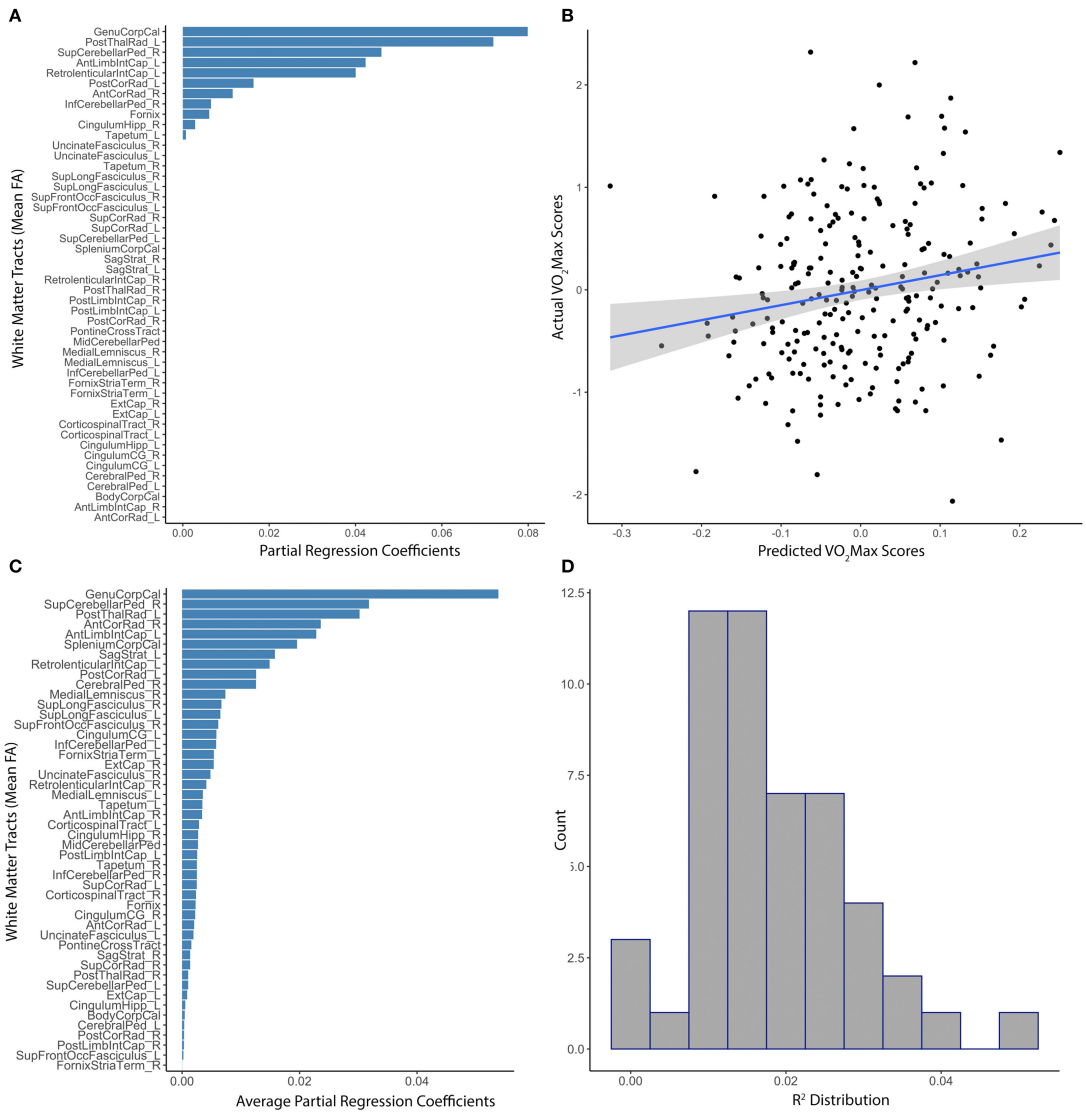
Distributed white matter tract integrity and cardiovascular fitness. **(A)** The absolute value of partial regression coefficients for each tract that remained non-zero after training the initial elastic net model predicting residualized VO_2_Max. **(B)** Scatterplot of the correlation between model-predicted and actual sex-adjusted estimated VO_2_Max (centered and scaled) within the set-aside test subset (*R*^2^ = 0.028, *p* = 0.003). **(C)** Absolute value of the average partial regression coefficients for each tract from all iterations of the ensemble modeling. **(D)** Distribution of *R*^2^s gathered from the subsequent ensemble modeling using 1,000 iterations of the elastic net model (Ensemble *R*^2^ = 0.013, 25th−75th quartile = 0.005–0.02).

White matter tract anisotropy predicted estimated VO_2_Max with initial model parameters of best fit of α = 0.44, λ = 0.66, MAE = 4.49, RMSE = 5.61. The absolute value of partial regression coefficients for multiple white matter tracts remained non-zero after training the initial elastic net model predicting VO_2_Max (Figure 2A). The correlation between model-predicted and actual VO_2_Max within the set-aside test subset was significant (*R*^2^ = 0.028, *p* = 0.003; Figure 2B). Subsequent testing confirmed the value of multiple white matter tracts in predicting VO_2_Max (Figure 2C). However, the distribution of *R*^2^ gathered from multiple iterations of the model through baggage testing indicated that the link between distributed white matter anisotropy and cardiovascular fitness was likely more modest than the initial estimations (Ensemble *R*^2^ = 0.013, median = 0.01, 25th−75th quartile = 0.005–0.02; Figure 2D). Of the individual white matter tracts included in the model that remained non-zero after training, some explained more unique variance than others. For example, the genu of the corpus callosum, the posterior thalamic radiata, the superior cerebellar peduncles, and parts of the internal capsule had the highest partial regression coefficients. Further, these tracts remained stable predictors throughout subsequent baggage testing (Figures 2A,C).

### Elastic Net Modeling of Healthy Lifestyle Behaviors (*N* = 854)

**Figure 3:**
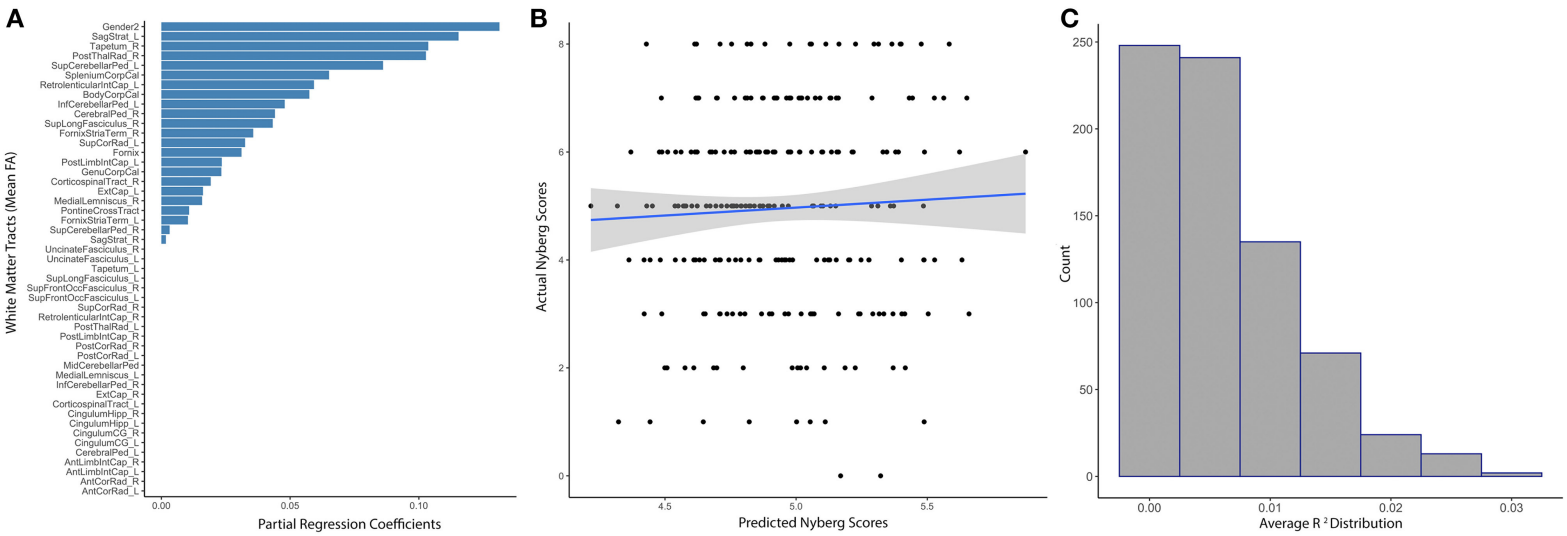
Distributed white matter tract integrity and Nyberg Health Index scores. **(A)** The absolute value of partial regression coefficients for each tract that remained non-zero after training the initial elastic net model predicting Nyberg Health Index scores. **(B)** Scatter plot showing the correlation between model-predicted and actual Nyberg Health Index scores (*R*^2^ = 0.0025, *p* = 0.44). **(C)** Distribution of *R*^2^s gathered from the subsequent ensemble modeling using 1,000 iterations of the elastic net model, which did not reveal any non-zero tracts (Ensemble *R*^2^ = 0.0062, 25th−75th quartile = 0.0001–0.008). Sex was included as a covariate in all modeling.

White matter tract anisotropy predicted estimated Nyberg Index Scores with initial model parameters of best fit of α = 0.56, λ = 6.26, MAE = 1.46, RMSE = 1.8. The absolute value of partial regression coefficients for multiple white matter tracts remained non-zero after training the initial elastic net model predicting Nyberg Health Index Scores (Figure 3A). Sex was the most relevant predictor of the Nyberg Health Index. The correlation between the model-predicted and actual Nyberg Health Index scores within the set-aside test subset were not significant (*R*^2^ = 0.0025, *p* = 0.44; Figure 3B). Subsequent baggage testing confirmed that the model lacked predictive utility (Ensemble *R*^2^ = 0.0062, median = 0.004, 25th−75th quartile = 0.0001–0.008, Figure 3C) and did not confirm the value of any white matter tracts in predicting scores.

## Discussion

Aging-related structural deterioration of the brain undergirds cognitive decline, one of the most debilitating symptoms of ADRD (Fjell et al., 2014; Vuksanovi et al., 2019; Elliott, 2020). The structural integrity of distributed white matter tracts is critical for normal cognition and age-related deterioration of white matter contributes to cognitive decline. Through the use of elastic net modeling, we found modest associations between cardiovascular fitness, as indexed by VO_2_Max, and the structural integrity of distributed white matter, as indexed by tract-wise FA, in a large population-representative birth cohort now in midlife. The observed associations between cardiovascular fitness and white matter integrity were distributed across the brain and remained stable predictors, though attenuated, through subsequent ensemble modeling. Emphasizing the importance of objective fitness measures, similar associations between healthy lifestyle behaviors, as indexed by the Nyberg Health Index, and white matter integrity were not observed.

Our discovery of associations between white matter integrity and cardiovascular fitness but not healthy lifestyle behaviors is consistent with research on exercise and structural integrity of the brain (Sexton et al., 2016; d'Arbeloff, 2020). Prior clinical trials have found that exercise interventions targeting age-related structural decline in gray matter only show positive results if the participants' cardiovascular fitness improved (d'Arbeloff, 2020). Similar findings have been reported in studies targeting white matter (Sexton et al., 2016). Thus, targeting improvements in healthy lifestyle behaviors to slow or prevent aging-related brain atrophy may not be effective if the interventions do not result in improved physiological fitness (d'Arbeloff, 2020). Moreover, links between gray matter and fitness emerged in prior studies regardless of the experimental condition suggesting that general improvement in cardiovascular fitness may have benefits for the brain (d'Arbeloff, 2020). Further research could help elucidate this pattern and help identify both the level of activity necessary to improve cardiovascular fitness and the threshold at which improvements in cardiovascular fitness may manifest in the brain. However, it is important to note that the Nyberg Health Index is based on self-report, which may introduce additional noise into analyses (Matthews et al., 2012). Thus, it is possible that objectively measured lifestyle behaviors may have a different association with distributed white matter integrity.

While our analyses leveraged information regarding the integrity of distributed white matter tracts to identify associations with cardiovascular fitness, further inspection revealed a differential contribution of some tracts over others to this overall effect. Specifically, tracts in frontal, temporal, and motor regions (i.e., corpus callosum, thalamic radiata, corona radiata, cerebellar peduncles, and internal capsule) accounted for greater unique variance in cardiovascular fitness in the elastic net model. This is consistent with some prior research on links between white matter, cardiovascular fitness, and aging. For example, the structural integrity of the cerebellum has been linked with reduced cognitive processing and general motor functioning in those who later develop ADRD (Toniolo et al., 2020). In addition, the integrity of white matter tracts in frontal and temporal regions may be particularly vulnerable to aging (Teipel et al., 2007; Wen et al., 2019; Toniolo et al., 2020), and lower structural integrity of these tracts has been linked with increased risk for cognitive decline and ADRD (Lee et al., 2015; Yang et al., 2016; Habes et al., 2018). For example, the corpus callosum, the tract that explained the most unique variance in our analyses, may be particularly relevant for age-related disorders including ADRD as it is highly involved in hemispheric integration and inhibition central to cognition (Goldman et al., 2017; Hsieh et al., 2020). Additionally, age-related decreases in FA within the genu of the corpus callosum has been linked with lower performance across a variety of cognitive domains such as working memory, executive function, and attention (Goldman et al., 2017; Loprinzi et al., 2020). Further, a recent meta-analysis found evidence suggesting aerobic exercise that enhances cardiovascular fitness is associated with increases in the structural integrity of the corpus callosum (Loprinzi et al., 2020).

Our study is not without limitations. First, the associations observed between tract-wise FA and VO_2_Max were quite small as was the overall variance explained. However, these effect sizes are from a population-representative sample with no selection bias and small effect sizes may be consequential over the long term, either because effects accumulate over time or because many individuals are affected (Funder and Ozer, 2019). Second, our data are cross-sectional as we have only one neuroimaging timepoint. In general, observational cross-sectional analyses are poor at leveraging causal information and establishing causal effect, in part due to a lack of randomization and variable control within study parameters and analyses. Third, our use of cross-sectional measures of both brain structure and cardiovascular fitness ignores the possibility of temporal trends within relationships. Given that aging-related decline occurs over time, future longitudinal studies should look to utilize multiple neuroimaging timepoints to assess the relationship between cardiovascular fitness and change in brain structure over time. Fourth, the Dunedin Study cohort is predominantly NZ European. Thus, replication is needed in diverse populations to identify how generalizable our findings may be across different demographics. Fifth, FA represents a summary measure of white matter integrity that lacks specificity in terms of which microstructural elements (e.g., axon density or degree of myelination) may be driving observed associations (Emsell et al., 2016). Future research could look to address this lack of specificity through the use of alternative white matter modeling techniques such as diffusion spectrum imaging or spherical deconvolution (Seunarine and Alexander, 2014). Sixth, our measure of healthy lifestyle behaviors was limited to alcohol consumption, tobacco smoking, physical inactivity, and obesity. Thus, it remains possible that other healthy lifestyle behaviors not tested here (e.g., sleep trends, blood pressure medication, etc.) may impact white matter integrity. Finally, while white matter integrity is strongly associated with cognitive ability and white matter atrophy with cognitive decline (Liu et al., 2017; Araque Caballero et al., 2018; Mito et al., 2018; Toniolo et al., 2020), how these associations interact with cardiovascular fitness must be explicitly tested in future research.

These limitations notwithstanding, our findings—though modest—suggest that the distributed integrity of white matter in midlife may serve as a useful target for in intervention studies leveraging positive changes in cardiovascular fitness. These findings are bolstered by the population-representative nature of our Study cohort who were all the same age when data was collected thereby removing age as a confound and increasing generalizability. Moreover, identifying links between cardiovascular fitness and white matter integrity at age 45 may be critical as midlife represents a point in the lifespan where aging-related structural decline is beginning to appear in the brain (d'Arbeloff et al., 2019) but irreversible damage may not have yet accrued. Thus, midlife may be an ideal period for early intervention and our findings lend further support to the specific potential of cardiovascular fitness interventions to slow or prevent aging-related decline in the brain.

## Data Availability Statement

The data analyzed in this study is subject to the following licenses/restrictions: The dataset reported in the current article is not publicly available due to lack of informed consent and ethical approval but is available on request by qualified scientists. Requests require a concept paper describing the purpose of data access, ethical approval at the applicant's institution, and provision for secure data access. We offer secure access on the Duke University and Otago University campuses. All data analysis scripts and results files are available for review. Requests to access these datasets should be directed to Terrie.Moffitt@Duke.edu.

## Ethics Statement

The studies involving human participants were reviewed and approved by Protocol: 2017-0593 C0683 The Dunedin Multidisciplinary Health and Development Study–Brain Imaging Sub-study Researcher(s): Caspi, Avshalom–Researcher Hariri, Ahmad–Researcher Moffitt, Terrie–Researcher Check-in Date: 10/14/2020 Renewal approved: September 18, 2019. The patients/participants provided their written informed consent to participate in this study.

## Author Contributions

TEM, AC, DI, SR, TRM, RP, AK, and AH designed research protocols. Td'A, AH, TEM, and AC performed and designed research concept. Td'A, AK, and MS analyzed data. Td'A and AH wrote the paper. All authors provided critical feedback and helped to shape the manuscript.

## Conflict of Interest

The authors declare that the research was conducted in the absence of any commercial or financial relationships that could be construed as a potential conflict of interest.
