# Supplementary material for: Midlife Cardiovascular Fitness Is Reflected in the Brain's White Matter
Source: Front Aging Neurosci. 2021 Apr 6;13:652575. doi: 10.3389/fnagi.2021.652575 (PMC8055854; doi:10.3389/fnagi.2021.652575)
Supplement: Supplementary file 1 [file Table_1.DOCX]

**Supplemental Information**

**Supplemental Table S1.** Average FA values (± standard deviation) for each white matter tract used in analyses.

| White Matter Tract | Mean FA (± SD) |
| --- | --- |
| Anterior Corona Radiata (Left) | 0.435 ± 0.023 |
| Anterior Corona Radiata (Right) | 0.437 ± 0.023 |
| Anterior Limb of Internal Capsule (Left) | 0.556 ± 0.20 |
| Anterior Limb of Internal Capsule (Right) | 0.554 ± 0.019 |
| Body of Corpus Callosum | 0.645 ± 0.028 |
| Cerebral Peduncle (Left) | 0.685 ± 0.024 |
| Cerebral Peduncle (Right) | 0.688 ± 0.026 |
| Cingulum – Cingulate Gyrus (Left) | 0.594 ± 0.029 |
| Cingulum – Cingulate Gyrus (Right) | 0.552 ± 0.028 |
| Cingulum – Hippocampus (Left) | 0.438 ± 0.034 |
| Cingulum – Hippocampus (Right) | 0.463 ± 0.037 |
| Corticospinal Tract (Left) | 0.536 ± 0.027 |
| Corticospinal Tract (Right) | 0.537 ± 0.029 |
| External Capsule (Left) | 0.443 ± 0.019 |
| External Capsule (Right) | 0.445 ± 0.020 |
| Fornix – Column & Body | 0.481 ± 0.042 |
| Fornix/Stria Terminalis (Left) | 0.508 ± 0.028 |
| Fornix/Stria Terminalis (Right) | 0.510 ± 0.028 |
| Genu of Corpus Callosum | 0.633 ± 0.028 |
| Inferior Cerebellar Peduncle (Left) | 0.503 ± 0.022 |
| Inferior Cerebellar Peduncle (Right) | 0.501 ± 0.024 |
| Medial Lemniscus (Left) | 0.558 ± 0.023 |
| Medial Lemniscus (Right) | 0.559 ± 0.022 |
| Middle Cerebellar Peduncle | 0.577 ± 0.017 |
| Pontine Crossing Tract | 0.463 ± 0.022 |
| Posterior Corona Radiata (Left) | 0.469 ± 0.021 |
| Posterior Corona Radiata (Right) | 0.473 ± 0.023 |
| Posterior Limb of Internal Capsule (Left) | 0.645 ± 0.019 |
| Posterior Limb of Internal Capsule (Right) | 0.654 ± 0.021 |
| Posterior Thalamic Radiata (Left) | 0.560 ± 0.025 |
| Posterior Thalamic Radiata (Right) | 0.563 ± 0.025 |
| Retrolenticular part of Internal Capsule (Left) | 0.566 ± 0.021 |
| Retrolenticular part of Internal Capsule (Right) | 0.563 ± 0.024 |
| Sagittal Stratum (Left) | 0.517 ± 0.023 |
| Sagittal Stratum (Right) | 0.524 ± 0.026 |
| Splenium of Corpus Callosum | 0.714 ± 0.019 |
| Superior Cerebellar Peduncle (Left) | 0.637 ± 0.025 |
| Superior Cerebellar Peduncle (Right) | 0.635 ± 0.025 |
| Superior Corona Radiata (Left) | 0.482 ± 0.021 |
| Superior Corona Radiata (Right) | 0.479 ± 0.021 |
| Superior Fronto-Occipital Fasciculus (Left) | 0.496 ± 0.027 |
| Superior Fronto-Occipital Fasciculus (Right) | 0.505 ± 0.025 |
| Superior Longitudinal Fasciculus (Left) | 0.485 ± 0.019 |
| Superior Longitudinal Fasciculus (Right) | 0.490 ± 0.021 |
| Tapetum (Left) | 0.549 ± 0.041 |
| Tapetum (Right) | 0.518 ± 0.045 |
| Uncinate Fasciculus (Left) | 0.527 ± 0.035 |
| Uncinate Fasciculus (Right) | 0.515 ± 0.039 |

**Supplemental Figure 1.** We conducted an attrition analysis using childhood neurocognitive functioning (the Wechsler Intelligence Scale for Children–Revised; WISC–R) and socioeconomic status (SES) to determine whether participants in the Phase 45 data collection were representative of the original cohort. a) No significant differences in WISC–R were found between the full cohort, those still alive, or those seen at Phase 45. Those who were deceased by the Phase 45 data collection had significantly lower scores on the WISC–R than those who were still alive (t=2.09, P=.04). b) No significant differences were found between the full cohort, those deceased, those alive, or those seen at Phase 45 on childhood SES.


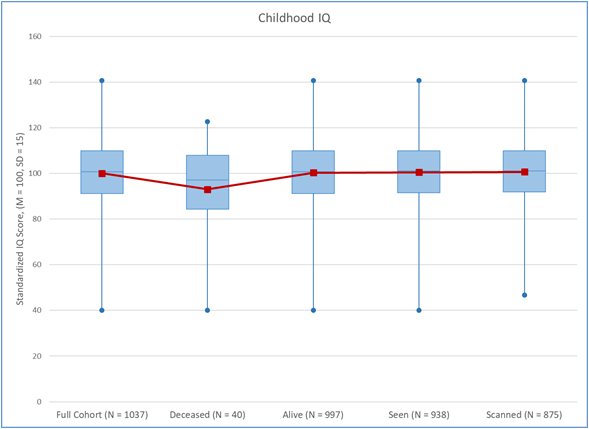


a

b


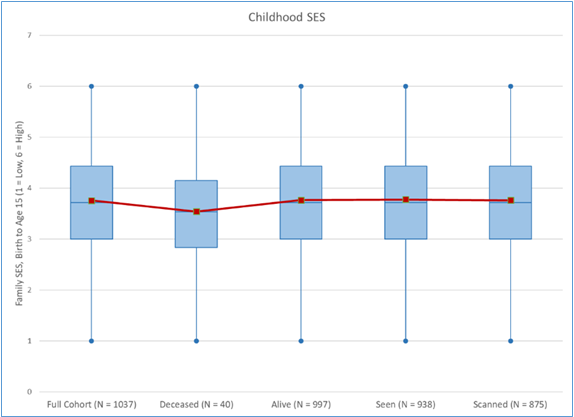


**Supplemental Figure 2**. Variable distributions of VO_2_Max (N = 801) and Nyberg Health Index scores (N= 854) in men and women. a) Men had an average VO_2_Max of 31.65 mL/min/kg and women had an average of 22.14 mL/min/kg. b) Men had an average Nyberg Health Index score of 4.8 and women had an average score of 5.12.

**a** **b**


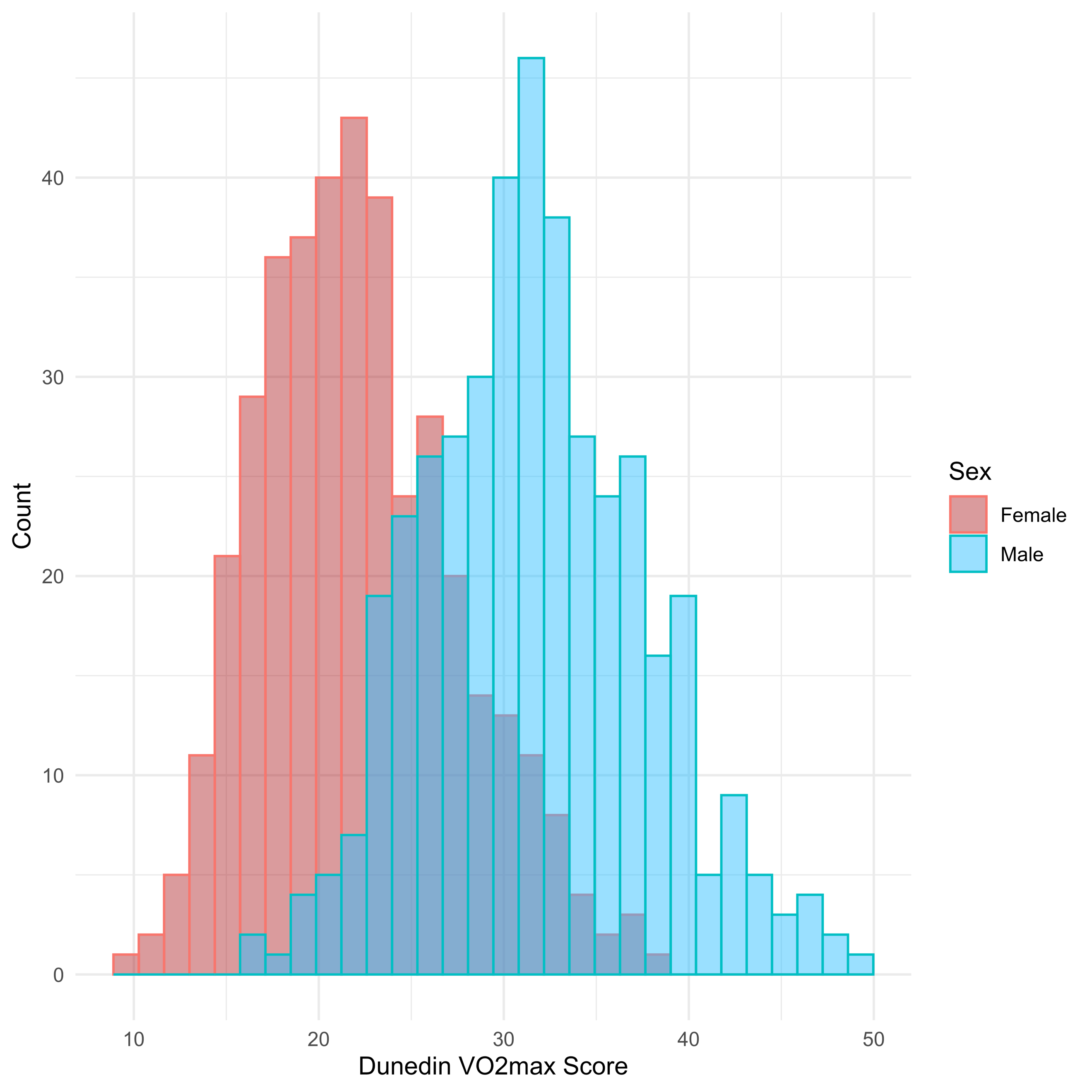

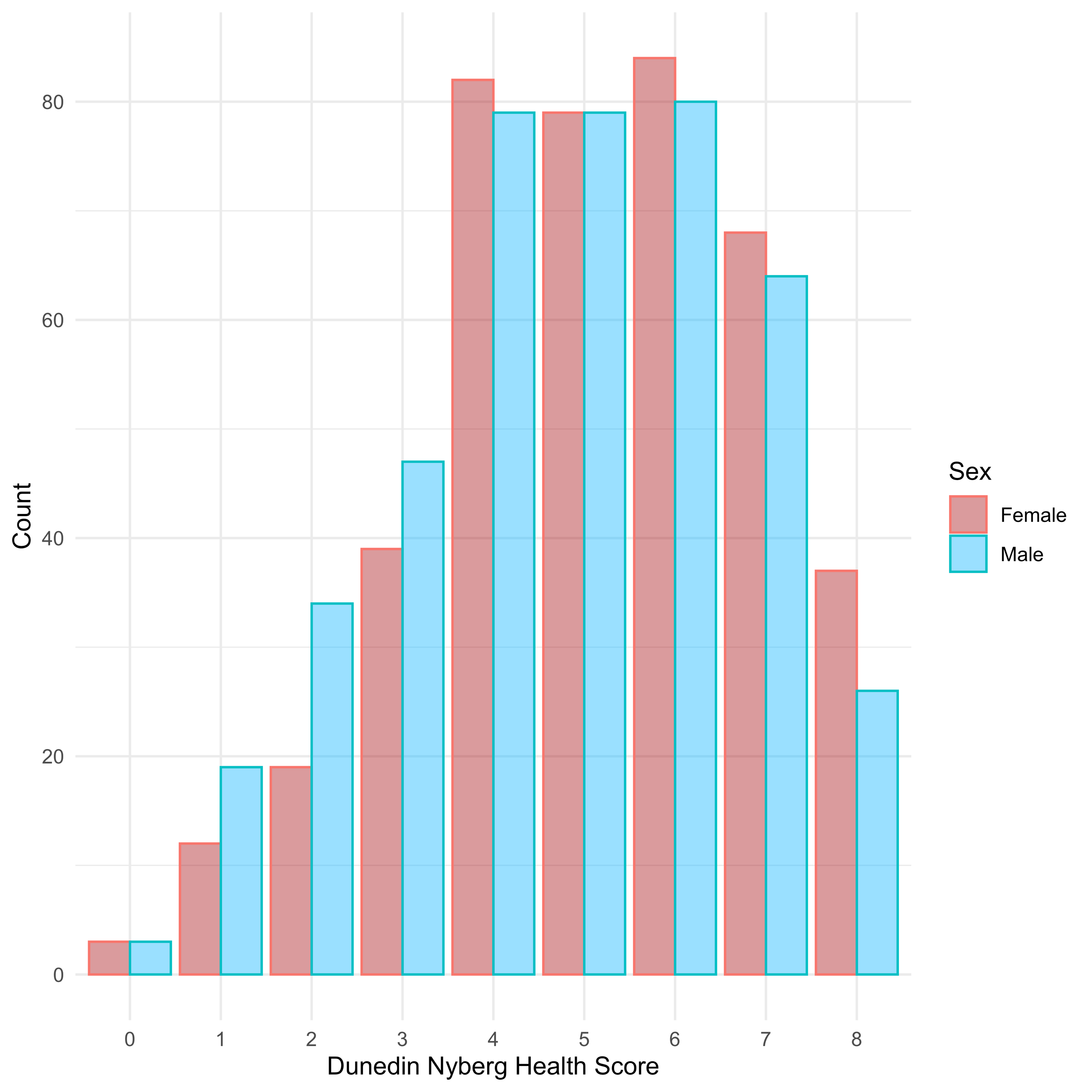


**

Supplemental Figure 3**. Heat map of correlations between all white matter tracts used in analyses.
